# Adherence to the ABCDE approach in relation to the method of instruction: a randomized controlled simulation study

**DOI:** 10.1186/s12873-021-00509-0

**Published:** 2021-10-15

**Authors:** Marjolein Linders, Mathijs Binkhorst, Jos M. T. Draaisma, Arno F. J. van Heijst, Marije Hogeveen

**Affiliations:** 1 Radboud Institute for Health Sciences, Department of Pediatrics, Radboud University Medical Center Amalia Children’s Hospital, Nijmegen, the Netherlands; 2 Department of Neonatology, Radboud University Medical Center Amalia Children’s Hospital, P.O. Box 9101, 6500 HB Nijmegen, the Netherlands

**Keywords:** Cardiopulmonary resuscitation, ABCDE approach, Education, Simulation training, Audiovisual aids

## Abstract

**Background:**

The Airway, Breathing, Circulation, Disability, and Exposure (ABCDE) approach is widely recommended and taught in many resuscitation courses. This study assessed the adherence to the ABCDE algorithm and whether this was affected by the instruction method used to teach this approach.

**Methods:**

Randomized controlled trial in which simulation was used as investigational method. Between June 2017 and January 2018, neonatal healthcare providers routinely participated in simulated neonatal advanced life support (NALS) scenarios, using a high-fidelity manikin. They were randomly assigned to a video-based instruction (intervention group) or a conventional lecture (control group) as the method of instruction. One blinded researcher evaluated the adherence to the ABCDE approach on video with an assessment tool specifically designed and tested for this study. The primary outcomes were: 1) the overall adherence and 2) the between-group difference in individual adherence to the ABCDE approach, both expressed as a percentage score. Secondary outcomes were: 1) the scores of each profession category (nurses, neonatal ward clinicians, fellows/neonatologists) and 2) the scores for the separate domains (A, B, C, D, and E) of the algorithm.

**Results:**

Seventy-two participants were assessed. Overall mean (SD) percentage score (i.e. overall adherence) was 31.5% (19.0). The video-based instruction group (28 participants) adhered better to the ABCDE approach than the lecture group (44 participants), with mean (SD) scores of 38.8% (18.7) and 27.8% (18.2), respectively (*p* = 0.026). The difference in adherence between both groups could mainly be attributed to differences in the adherence to domain B (*p* = 0.023) and C (*p* = 0.007). Neonatal ward clinicians (39.9% (18.2)) showed better adherence than nurses (25.0% (15.2)), independent of the study group (*p* = 0.010).

**Conclusions:**

Overall adherence to the ABCDE algorithm was rather low. Video-based instruction resulted in better adherence to the ABCDE approach during NALS training than lecturing.

**Trial registration:**

ISRCTN registry, trial ID ISRCTN95998973, retrospectively registered on October 13th, 2020.

**Supplementary Information:**

The online version contains supplementary material available at 10.1186/s12873-021-00509-0.

## Background

The systematic ABCDE approach, acronym for Airway, Breathing, Circulation, Disability, and Exposure, is a widely accepted, expert-based algorithm for the management of (possibly) critically ill or injured patients of all age categories [1, 2]. The ABCDE approach functions as an assessment algorithm, which enables healthcare providers to identify and respond to critical conditions in order of priority [2, 3]. Experts believe that the ABCDE approach may improve the assessment and initial treatment of those in need of emergency care [1, 2]. Therefore, (inter)national guidelines recommend the use of this approach whenever serious illness or injury is suspected, regardless of the underlying cause [1, 2, 4].

Although the ABCDE approach is ubiquitously advocated, personal observations and limited data from previous research indicate that adherence to the ABCDE approach needs improvement, both during simulation training and in clinical care [5]. There are multiple strategies conceivable to improve algorithm/guideline adherence. For example: the algorithm’s feasibility and scientific base may be enhanced, consensus on algorithm application may be augmented within teams, and various mnemonics, checklists, prompts, feedback devices, and other adjuncts may be used [6]. Yet another straightforward strategy is to develop effective training programs, through which healthcare professionals can acquire and retain the knowledge and skills required to apply the algorithm adequately.

Video-based instruction (VBI) has been used for the education of resuscitation skills. It is an attractive instructional method, for it combines the advantages of observational learning and audiovisual support, it always shows a perfect demonstration, and it could be more cost-effective and less time-consuming than conventional teaching. Several studies found promising results for VBI, including improved practical skills and increased self-confidence [7–10]. However, the majority of these studies used an instructional video as part of a self-directed learning approach instead of an in-hospital course [11, 12]. Furthermore, evidence regarding the most effective instruction method for teaching the ABCDE approach in particular is virtually absent.

The current study was the first step of a more elaborate project investigating adherence to the ABCDE approach and ways to improve this adherence. Simulation was used as a methodology in this randomized controlled trial to investigate overall adherence and the possible difference in individual adherence to the ABCDE algorithm between neonatal healthcare professionals who received either VBI or a conventional lecture (CL) (primary outcomes). The adherence of each profession category (nurses, neonatal ward clinicians, fellows/neonatologists) and the adherence to the separate domains (A, B, C, D, and E) of the algorithm were also evaluated, independent of the study groups (secondary outcomes).

## Methods

A randomized controlled, single-blinded study was conducted in the simulation facility of a level III perinatal care center (Radboud University Medical Center Amalia Children’s Hospital, Nijmegen, the Netherlands) between June 12th, 2017 and January 24th, 2018. The reason for targeting the neonatal clinical population was practical convenience, since the principal investigators were neonatologists and existing obligatory training programs concerning the ABCDE approach in neonatal advanced life support (NALS) could be used. This study was approved by the Institutional Review Board of the Radboud University Medical Center (file number 2017–3513) and reported based on established guidelines for simulation-based research, which are extensions to the Consolidated Standards of Reporting Trials (CONSORT) statement [13].

### Study participants

Nurses, nurse practitioners (NP), physician assistants (PA), pediatric residents, neonatal fellows, and neonatologists employed in the neonatal intensive care (NICU) and high care units of the Radboud medical center participated. In as much as NP, PA, and pediatric residents performed the same work on the ward, they were collectively referred to as neonatal ward clinicians. Participants were automatically recruited for this study, since they had to take part in periodic neonatal advanced life support (NALS) simulation training in this center. The only additional eligibility criterion was the availability to participate in the training program during the study period. The exclusion criterion was refusal to give informed consent to use the video recordings of the simulation scenarios for research purposes. This did not occur; written informed consent was obtained from all participants. The eventual number of participants depended on the number of trainings given within the time frame of the study and the attendance rate of the healthcare professionals. Background characteristics were collected with a short questionnaire (Additional file 1).

### Study design

Simulations of an existing training program were used, since scheduling separate simulations exclusively for this study was not possible. The latter would interfere too much with the usual activities of the simulation center, and it was expected that a limited number of healthcare professionals would be able to attend these additional, study-related simulations. On training days, once or twice a month, the instruction method for that day’s team of eight nurses, one neonatal ward clinician, and one neonatal fellow/neonatologist was randomly allocated using sequentially numbered opaque sealed envelopes (SNOSE). Randomization was performed by a person not involved in the study, with an allocation ratio of 1:1. The intervention group was trained using VBI, the control group was trained using a CL. Each training day lasted eight hours. Except for the instruction method, training days were identical in both groups. Participants were asked not to inform future participants about the contents and proceedings of the training.

### Instruction methods

The instructional video (15 min) showed a NICU nurse and senior pediatric resident consecutively performing perfect demonstrations of the ABCDE approach during a NALS simulation scenario. The video was interspersed with brief screenshots with text emphasizing the tasks performed for the various domains of the algorithm. The video was specifically made for this study by a neonatologist with experience in simulation-based training (MB). The lecture (20–30 min) concerned a PowerPoint presentation of 30 slides (Microsoft Office PowerPoint 2007), in which the application of the ABCDE approach during NALS was explained step-by-step. The lecture was given by an experienced simulation specialist, the video was shown without additional comments. Questions of the participants were only briefly answered to elucidate the content of the lecture or video, without digressions. Both lecture and video were presented once, and they were only available on training days, not online or elsewhere. MB meticulously checked that the video and lecture contained the same teaching content.

### Equipment and manikin

After instruction of the ABCDE approach, participants received a 15 min orientation to the simulation environment, equipment, and manikin by the simulation operator. The simulation room closely mimicked the clinical work environment, including a patient bed, monitor, ventilator, T-piece oxygen delivery device, suction, intravenous setup, defibrillator, and crash cart with basically the same equipment and medications as available on the ward (Table 1). All equipment had standardized locations within the simulation room and was carefully demonstrated. The phone (to call for back-up assistance) and intercom (to communicate with the simulation operator) were introduced to the participants. All features, possibilities (e.g. vascular access, umbilical cord catheterization, chest drain placement, tracheal intubation, pupil reflexes, and clonic seizures), and impossibilities (e.g. changes in skin color and temperature, occurrence of skin abnormalities, chest retractions, and capillary refill time) of the manikin were clarified. An originally low-fidelity manikin (Newborn Anne, Laerdal Benelux, Amersfoort, the Netherlands) was used, which had been equipped with various features (mechanics to enable active breathing; internal speakers for crying, grunting, breath, and heart sounds) and recording capabilities (magnetic switch to measure compression depth; pressure sensor to assess hand placement, compression rate, and recoil; and flow sensor to quantify tidal volumes) by a technical simulation expert (Tim Antonius), transforming it into a high-fidelity manikin. Tidal volumes, airway pressures, and chest compression characteristics had been calibrated beforehand to ensure reliable mechanical measurements.

**Table 1:** Equipment, situation, and scenario for the simulations

**Equipment**	
• High-fidelity manikin • Standard NICU equipment: endotracheal tubes, laryngoscope, Magill forceps, suction catheters, IV cannulas, syringes, chest drains, stethoscope, medications, parenteral solutions, simulated blood products • T-piece oxygen delivery device (Neopuff™ Infant Resuscitator) and ventilator • Standard patient monitor • Defibrillator with neonatal pads • Audio equipment (intercom and phone) • Video equipment (3 HD cameras)	
**Situation**	
• Orientation to the manikin, room, and equipment by the simulation operator • Introduction of the patient to two nurses starting the scenario • One ward clinician and one fellow/neonatologist standby outside the room • Remaining participants (nurses) in an adjacent room with live stream connection • Simulation operator^a^ in a control room behind a one-way mirror, available via intercom	
**Scenario**	
• Two nurses start their ABCDE assessment when the neonate deteriorates • Back-up assistance on request: ward clinician is summoned and starts ABCDE assessment • Back-up assistance on request: fellow/neonatologist is summoned and starts ABCDE assessment • Brief answers to questions by the simulation operator; no other cues or suggestions • All 3 profession categories must have finished their assessment before scenario ends	

*HD* high-definition, *IV* intravenous, *NICU* neonatal intensive care unit

^a^Two simulation operators alternately supervised the scenarios. Both operators are senior consultants with more than 10 years of experience in neonatology and simulation-based education and research; both have medical education qualifications; both completed the EuSim Simulation Instructor Course. A highly experienced nurse specialized in neonatal simulation assisted the simulation operators on every training day

### Training sessions

Subsequently, the team participated in four to five NALS scenarios. Each scenario was run by two different nurses, the same ward clinician, and the same fellow/neonatologist. The remaining participants (6 nurses) observed the performance in the adjacent briefing/debriefing room through a live audiovisual connection. Scenarios were controlled by a simulation operator from behind a one-way mirror. All scenarios started with the two nurses. This was usual practice in simulation training in this center. It mirrored clinical reality, in which a nurse instantaneously asked a colleague for help when a patient showed aberrant vital signs, so they performed the initial assessment together. When the situation deteriorated, the nurses summoned the ward clinician, who almost invariably called for back-up assistance by the fellow/neonatologist. Scenarios lasted approximately 15 min, during which all three profession categories had to perform a systematic ABCDE approach. The nurses performed the first ABCDE assessment when the newborn started to deteriorate. The ward clinician and fellow/neonatologist performed the second and third ABCDE assessments, respectively, upon their arrival at the scene. During assessment, the principle of a ‘sterile cockpit’ was applied. Notes, pocket cards, or other adjuncts were not permitted, because the aim was to investigate the effect of the instruction method on adherence in isolation. Otherwise, it would have been very difficult to control for differences in the use of these adjuncts. Each scenario was immediately followed by a non-scripted, video-assisted, operator-led debriefing (30 min) in the debriefing room with all participants, including the observing nurses [14]. Formative feedback was provided, not only on the ABCDE assessment, but also on other aspects, such as skill performance, clinical reasoning, differential diagnosis, and crew resource management principles. Debriefers were unaware of group assignment.

### Scenarios and video recording

Various patient scenarios were used (e.g. apnea, arrhythmia, sepsis, seizures, and metabolic derangements). Scenarios involving resuscitation at birth were also part of the existing training program. The medical staff of the department considered it undesirable to discontinue these birthing scenarios for the duration of the study (> 6 months). Consequently, these scenarios did take place, but were excluded from the study, for they require another algorithm. The scenarios were semi-structured, with scenario progression according to prespecified triggering events. All scenarios were videotaped using three high-definition cameras: one on the manikin’s side, one affixed to the overhead radiant warmer, and one ceiling-mounted overview camera. These views were combined with the vitals from the monitor into one 4-screen window for assessment.

### Development of assessment tool

A suitable assessment tool to score adherence to the ABCDE algorithm could not be found. The assessment form developed by Drost-de Klerck et al. was also not compatible with the scoring procedure, for it was based on the slightly different ABCDE structure of the (adult) Advanced Life Support [15]. Therefore, a novel assessment instrument with a conventional, trichotomous scoring system was developed by MB and MH and applied in this study. All 24 items of the ABCDE approach, as presented in the Dutch Advanced Pediatric Life Support (APLS) course manual [4], were incorporated and categorized under the appropriate domains (A, B, C, D, and E) (Additional file 2). Note that the Dutch NALS course manual was not issued yet at the time of this study. For all items, 2, 1, and 0 points were awarded for adequate and timely performance, for incomplete or out-of-sequence performance, and for inadequate and out-of-sequence performance, respectively. Detailed scoring instructions were formulated. Each participant could receive a maximum score of 48 points. Items were classified as ‘not assessable’ whenever participants could not finish their ABCDE approach, because they rapidly summoned extra help. Such a situation was strictly defined in the scoring instructions. The final score for individuals’ adherence to the ABCDE algorithm was expressed as a percentage score and calculated by dividing the number of awarded points by the number of points for all assessable items, multiplied by 100%.

Prior to the actual study, the assessment tool was tested for intra-observer reliability. Ten previously videotaped simulation scenarios were assessed twice by the same observer (ML) with an interval of 2 weeks to prevent recall of ratings [16]. The intraclass correlation coefficient (ICC) was 0.87 (95% CI 0.74–0.94), with an overall difference of 1.4% (*p* = 0.39), which signifies an almost perfect agreement. Assessment of inter-observer reliability was not needed, for all videos were rated by the same observer (ML). Face validity was established, since consensus on the assessment instrument was reached among 6 experts in the fields of neonatal and pediatric life support. Content validity was ensured, because all items were derived from the Dutch APLS course manual.

### Assessment

All videos were assessed several weeks later by one researcher (ML), who was specifically trained in video-based scoring of the ABCDE approach in NALS scenarios through elaborate instruction by the designers of the assessment instrument and by means of proof scoring videos from earlier training sessions. ML was blinded to the received instruction method; she was not present during the scenarios. Only primary ABCDE assessments were scored, not the re-assessments after interventions. In as much as the two nurses worked closely together, their performance was combined and they were scored as one healthcare professional.

### Statistical analysis

A sample size calculation was performed to estimate the number of participants needed to achieve a power of 80% with a statistical significance of 0.05 (two-tailed) for the primary outcome (between-group difference in adherence). Twenty-one participants per group were required for an expected realistic and clinically relevant difference of 50% of the percentage score in the intervention group compared to the control group. The standard deviation used in the sample size calculation (16%) was obtained by assessing 31 previously videotaped ABCDE approaches performed during earlier NALS simulation scenarios.

Statistical analyses were performed using SPSS (IBM SPSS Statistics 22 for Windows, Armonk, NY, US) and SAS 9.4. Variables were expressed as means (SD) or medians (IQR), as appropriate. The unit of analysis was the ABCDE assessment of the individual participant. Background characteristics were analyzed with the Fisher’s exact test, Chi-Square test, and Mann-Whitney U test. Unpaired T-tests were used to assess the normally distributed primary outcomes. Subanalyses were carried out for the three profession categories, using Analysis of Variance (ANOVA), with post-hoc analysis by Tukey’s test. Differences in the performance on the domains of the ABCDE algorithm were determined by Mann-Whitney U tests. A *p*-value < 0.05 was considered statistically significant.

## Results

**Fig. 1:**
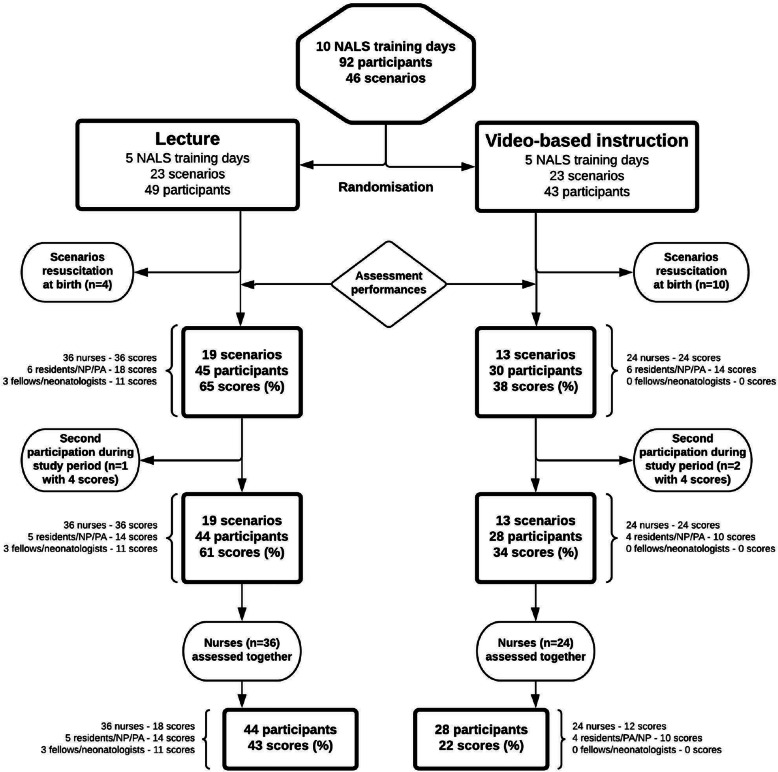
Flowchart of participant inclusion

Background characteristics, including previous experience with NALS simulation, were similar in both groups (Table 2). Ninety-two neonatal healthcare professionals participated on 10 NALS training days (Fig. 1). The attendance rate of fellows/neonatologists was unfortunately limited due to their clinical obligations. Fourteen of the 46 scenarios involved resuscitation at birth; these were excluded, as described above. Thirty-two scenarios, in which 75 neonatal healthcare professionals participated, were assessed, resulting in 103 percentage scores. One resident and two PA participated on two separate days during the study period; their last participation (2 in the VBI group, 1 in the CL group) was excluded. As said, the two nurses of each scenario were assessed as one provider. Eventually, 65 percentage scores were available for analysis. The VBI group consisted of 28 participants with 22 percentage scores, the CL group of 44 participants with 43 percentage scores. The fact that group sizes were eventually different in spite of a 1:1 randomization was mainly caused by the exclusion of more birth scenarios in the VBI group.

**Table 2:** Background characteristics

Characteristic	CL ^**a**^		VBI		***p*** value ^**b**^
Number of participants, n (%)	44		28		0.358 ^c^
Nurses	36	(81.8)	24	(85.7)	
Pediatric residents/NP/PA	5	(11.4)	4	(14.3)	
Neonatal fellows/neonatologists	3	(6.8)	0	(0.0)	
Age, median years (IQR)	44	(30.0–56.8)	42	(37.5–52.8)	0.871 ^d^
Sex, n of women (%)	40	(90.9)	26	(92.9)	1.000
Previous participation in NALS training, n (%)					0.608
< 4 times	16	(36.4)	8	(28.6)	
≥4 times	27	(61.4)	20	(71.4)	
Participation in relevant courses, n (%) ^e^					0.332
Yes	21	(47.7)	10	(35.7)	
No	22	(50.0)	18	(64.3)	
Experienced real-life neonatal resuscitation, n (%)					0.778
≤5 times	30	(68.2)	21	(75.0)	
> 5 times	13	(29.5)	7	(25.0)	
Working experience in pediatrics, n (%)					0.585
≤5 years	13	(29.5)	6	(21.4)	
> 5 years	31	(70.5)	22	(78.6)	
Working experience at neonatal IC or HC, n (%)					1.000
≤2 years	10	(22.7)	6	(21.4)	
> 2 years	34	(77.3)	22	(78.6)	
Working experience at pediatric IC or HC, n (%)					0.572
≤2 years	34	(77.3)	20	(71.4)	
> 2 years	9	(20.5)	8	(28.6)	

*CL* conventional lecture, *EPLS* European Paediatric Life Support, *HC* high care, *IC* intensive care, *IQR* interquartile range, *NALS* neonatal advanced life support, *NLS* neonatal life support, *NP* nurse practitioner, *PA* physician assistant, *PALS* pediatric advanced life support, *VBI* video-based instruction

^a^Some background information of one participant in this group could not be retrieved

^b^Group differences were tested with the Fisher’s exact test, unless stated otherwise

^c^Group difference regarding profession category was tested with the Chi-Square test

^d^Group difference regarding age was tested with the Mann-Whitney U test

^e^e.g. NLS/NALS, PALS, EPLS

**Fig. 2:**
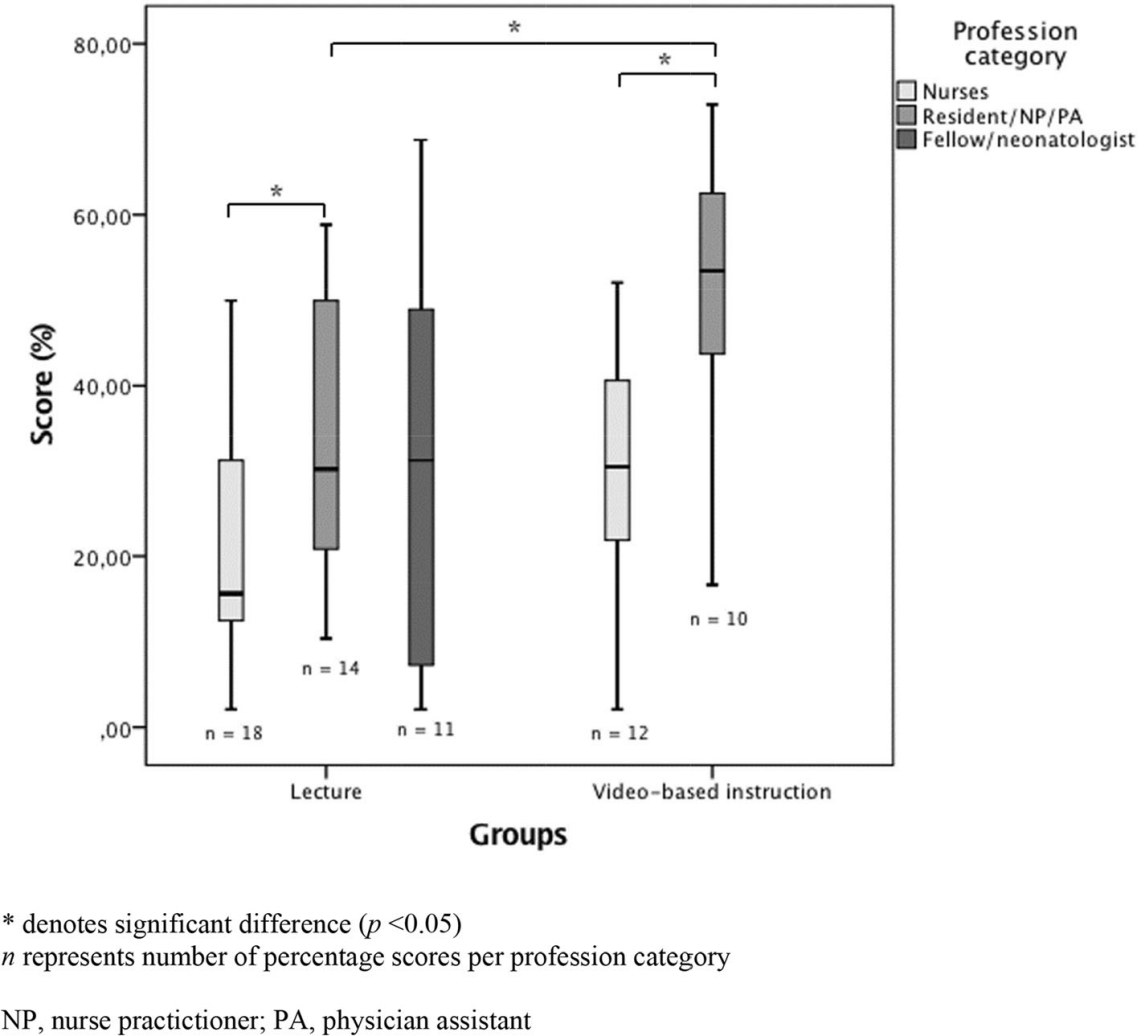
Percentage scores per profession category in both groups

All items in all scenarios were visible/audible, there were no missing data due to audiovisual shortcomings. The overall mean percentage score (SD), reflecting overall adherence to the ABCDE algorithm, was 31.5% (19.0). The VBI group showed better adherence than the CL group, with mean percentage scores (SD) of 38.8% (18.7) and 27.8% (18.2), respectively (*p* = 0.026) (Table 3). This difference remained significant when, in addition to the 1 resident and 2 PA participating for the second time, the potentially biased team members of these scenarios were also left out from analysis (data not shown). Subgroup analysis showed that the adherence of nurses and ward clinicians was better in the VBI group compared to the CL group, with a significant difference in percentage scores between the ward clinicians of the two groups (49.2% (17.3) vs. 33.3% (16.3), respectively) (*p* = 0.031) (Fig. 2, Table 3). Comparison of the performance of fellows/neonatologists was impossible, since the VBI group did not include fellows/neonatologists. A difference in performance among the profession categories was found (*p* = 0.013), with a significantly better adherence in ward clinicians compared to the nurses, both in the VBI group (*p =* 0.013), the CL group (*p* = 0.039), and independent of the study groups (*p* = 0.010) (Fig. 2). Participants’ adherence to the domains of the ABCDE algorithm is presented in Table 4. The VBI group scored higher than the CL group on all domains, with a significant difference in domains B and C.

**Table 3:** Adherence to the ABCDE algorithm

Profession category	Percentage score ^**a**^						
	Overall		CL		VBI		***p*** value
	n	Mean score (%) (SD)	n	Mean score (%) (SD)	n	Mean score (%) (SD)	
**Nurses**	30	25.0 (15.2)	18	21.5 (14.4)	12	30.1 (15.6)	0.135
**Residents/NP/PA**	24	39.9 (18.2)	14	33.3 (16.3)	10	49.2 (17.3)	**0.031**
**Fellows/neonatologists**	11	31.1 (23.8)	11	31.1 (23.8)	0	–	–
**All combined**	65	31.5 (19.0)	43	27.8 (18.2)	22	38.8 (18.7)	**0.026**

*CL* conventional lecture, *NP* nurse practitioner, *PA* physician assistant, *VBI* video-based instruction.

^a^Analyses are based on the number of percentage scores. Adherence to the ABCDE algorithm was analyzed with the unpaired T-test, since the data were normally distributed

**Table 4:** Adherence to the domains of the ABCDE algorithm

Domains	Percentage scores for domains of the ABCDE algorithm ^**a**^							
	Overall			CL		VBI		
	Maximum score, n (%)	Median score ^b^ (%) (IQR)		Median score ^b^ (%) (IQR)		Median score ^b^ (%) (IQR)		*p* value^b^
**ABCDE**	48 (100)	31.3	(14.6–49.0)	25.0	(12.5–43.8)	40.6	(24.5–53.1)	**0.025**
**A: Airway**	2 (100)	100.0	(50.0–100.0)	50.0	(0.00–100.0)	100.0	(50.0–100.0)	0.135
**B: Breathing**	20 (100)	35.0	(15.0–55.0)	25.0	(15.0–50.0)	47.5	(25.0–60.6)	**0.023**
**C: Circulation**	12 (100)	33.3	(16.7–62.5)	25.0	(8.30–50.0)	50.0	(33.3–66.7)	**0.007**
**D: Disability**	10 (100)	10.0	(0.00–20.0)	0.00	(0.00–10.0)	15.0	(0.00–20.0)	0.215
**E: Exposure**	4 (100)	0.00	(0.00–50.0)	0.00	(0.00–50.0)	25.0	(0.00–50.0)	0.114

*CL* conventional lecture, *VBI* video-based instruction

^a^Analyses are based on the number of percentage scores

^b^Analyzed using a non-parametric test (Mann-Whitney U) and displayed in median (IQR) due to the non-normal distribution of the percentage scores within the domains

## Discussion

This study shows that the adherence to the ABCDE approach by neonatal healthcare professionals during simulated NALS scenarios improved when the ABCDE approach was taught with video-based instruction (VBI) instead of a conventional lecture (CL). However, the overall adherence to the ABCDE approach was quite low, emphasizing the need for continuing education.

Although several studies have previously looked at the ABCDE approach, only a few of them focused on the ABCDE approach as primary outcome. These studies were not comparable to this study due to differences in setting (e.g. prehospital) and/or outcome (e.g. time to completeness instead of adherence) [17, 18]. So far, no research has been conducted evaluating video-based instruction in teaching the ABCDE approach. Merely one study was found that specifically evaluated the performance of the ABCDE approach in clinical practice [5]. In an emergency department setting, Olgers et al. showed that, *when used*, the ABCDE approach was done highly complete (mean performance of 83.5% of needed items) with a median duration of 7 min. This outcome seemed to contrast the rather low adherence in this study. However, in the study by Olgers et al.*,* participants (all physicians) could *choose to be observed*, which may have caused bias as well as the Hawthorne effect (i.e. improved performance due to the awareness of being observed). Also, their participants had completed a two-day course on the ABCDE approach prior to the study. In this study, *all* participants (including non-physicians) of an existing training program were observed, they were less aware of being specifically observed for the ABCDE approach, and they received an instruction of merely 15–30 min. Furthermore, 7 min was rather long to complete an ABCDE assessment. Based on the most completely performed ABCDE approaches in this study, the impression was that the approach can be accomplished in 2–3 min. In addition, the authors did not provide information on the reliability and validity of their assessment tool, nor did they provide scoring instructions, which made it difficult to interpret their results. A remarkable finding in their study was that the ABCDE approach was *not used* in 67% of (potentially) unstable patients.

The difference in adherence between VBI and CL could be attributed to various factors. First, observational learning (i.e. an instructional video) increases self-efficacy, raising the probability that providers will use their knowledge and skills in an emergency situation [19]. Second, the instructional video may have led to better adherence, because all items, domains, and key messages regarding the use of the ABCDE algorithm were repeated several times in the video, and repetition is a generally accepted learning principle. Third, the demonstrations in the video were performed by peers. Since peers are ‘models’ that are demographically and psychosocially similar to the learners, their instructions will likely conduce to more effective learning [19]. At last, it is generally believed that transfer of knowledge and skills is enhanced when supported with audiovisual means.

Besides improving the adherence to the ABCDE algorithm, VBI can have additional benefits compared to other instructional methods, such as lectures and live instructor-led demonstrations. VBI can be more cost-effective, for expensive instructors can be partially replaced by an instructional video. It can also be less time-consuming, because videos can be watched by the participants at any moment, even prior to the actual course. Moreover, VBI can be standardized and always shows a perfect demonstration, in contrast to a live demonstration.

The reason for choosing the individual participant as the unit of analysis, instead of focusing on team performance, was threefold: 1) In actual practice –especially on clinical wards– the ABCDE approach is often not performed in a fixed team, that is fully present at the moment a patient starts to deteriorate. Instead, a more dynamic process is usually involved, in which a team gradually assembles as additional professionals are summoned to the scene for extra support. This sequential process requires consecutive ABCDE assessments, also because the patient’s condition and team leadership may change over time. Therefore, it seemed most appropriate to study the consecutive assessments of the individual providers to match the clinical situation. Parenthetically, one may infer from this study that all team members had to perform an ABCDE approach. This was, however, not true, but only appeared to be so due to the small size of the teams in this study. Also, even though the assessment focused on the professional who was responsible for the ABCDE evaluation at a specific moment during the scenario, this person could certainly be informed about relevant patient characteristics by his/her team members; 2) From an educational point of view, it was considered beneficial for the learning process to repeat the ABCDE assessment several times during the training sessions; 3) From a statistical perspective, choosing the team as the unit of analysis would have been troublesome. For one thing, it would have been very difficult to define and compare the background characteristics of the various teams.

This study was performed in a simulation environment. We nevertheless believe that the results can be generalized to the clinical setting. After all, a high-fidelity manikin, a room with high environmental fidelity, and realistic scenarios with clinically relevant neonatal morbidities evolving in a physiologically accurate manner were used. The interactions among and emotions of the participants closely resembled the clinical situation. The fact that a variety of semi-structured scenarios was used favors transferability of findings to the clinical context. The assessment instrument is also suitable for clinical patients. Although mean adherence in the VBI group was ‘only’ 38.8%, the difference of 11% compared to the CL group may be clinically relevant. A mean improvement of 11% equals an increase of 5 points on the assessment tool, which implies 3–5 additionally performed items (out of a total of 24 items). In clinical practice, these additionally performed items may very well lead to a better assessment of the patient. VBI should therefore be regarded as superior to conventional lecturing for teaching the ABCDE approach. Our suggestion would be to incorporate VBI in the educational arsenal of resuscitation courses.

The ABCDE approach is well-known and widely used. Still, its reputation is not completely flawless. Some shortcomings of this approach have to be acknowledged. When one carefully compares the components of the ABCDE algorithms as described in the manuals of the various types of life support, it becomes apparent that the ABCDE approach is not really universal. There are several subtle differences among the ABCDE evaluations of the APLS, European Paediatric Advanced Life Support, Advanced Life Support, Advanced Trauma Life Support, et cetera. One of our future goals is to investigate whether it is useful and achievable to create a truly universal ABCDE algorithm, which might facilitate professionals in treating patients who would normally be ‘out of their comfort zone’. Furthermore, the ABCDE approach is not universally accepted. Some healthcare professionals consider it too elaborate and rigid, while others think it misses important clinical information (unpublished data). The approach also lacks a firm evidence base in terms of effectiveness and benefit for patient outcomes [12]. On the other hand, a structured approach, such as the ABCDE algorithm, helps healthcare professionals to focus on the most life-threatening problems and guides initial treatment choices [12]. It also ensures that resuscitation team members ‘speak the same language’. In other words, the ABCDE approach can be seen as the ‘lingua franca’ of emergency medicine. Deviations from this algorithm may be needed in certain circumstances. Nonetheless, the ABCDE algorithm is the prevailing, expert-based approach to critically ill patients. It seems prudent to abide by this algorithm as much as possible, as stated in the international guidelines.

### Strengths

The main strengths of this study were the randomization of participants to prevent selection bias, blinding of the video assessor to the intervention, and the fact that the participants were not fully aware of the specific aim of this study, since they gave informed consent for research purposes in general. An assessment tool with face and content validity and high intra-observer reliability was used, substantiating the validity and reliability of the results. Sufficient power for the primary outcome was reached. The conclusion that VBI is superior to CL in terms of adherence to the ABCDE algorithm was not only corroborated by the overall between-group difference, but also by the results on the individual domains.

### Limitations

In addition to the considerations mentioned above regarding generalizability of this study and the imperfections of the ABCDE algorithm in general, some limitations arose while conducting this study. Most limitations were related to the use of an existing training program. The two nurses were assessed as one despite possibly different background characteristics. The training sessions took place during regular day shifts. As a result, fellows/neonatologists were sometimes unable to attend the simulations, since urgent matters demanded their presence on the ward. This explained the limited inclusion of these healthcare professionals and precluded conclusions about their adherence. It was not possible to compare both groups at baseline regarding their adherence to the ABCDE algorithm. However, considering their similar background characteristics, the two groups were probably not importantly divergent in this regard. Since participants were randomly assigned to either the intervention or control group, possible confounding factors were probably divided equally over both groups and therefore did not substantially influence the primary outcome of this study. In as much as physicians (i.e. ward clinicians and fellows/neonatologists) participated in multiple scenarios, they may have benefitted from a learning curve. This learning curve did probably not affect the difference in adherence between the VBI and CL groups, because, if it occurred, it did so in both groups. However, it may have influenced the comparison between nurses and physicians, because nurses only performed one scenario and could therefore benefit less from a learning curve. Support for this may be found in comparing nurses and physicians only in their first scenario, since this comparison does not show a significant difference. However, these results could not be reliably interpreted, because the study was underpowered for such a subanalysis.

### Future research

Further studies into this subject matter are highly recommended. Some follow-up studies are already planned by our research group, including evaluation of adherence to the ABCDE approach in the clinical situation. Additional research is needed to find out which (bundle of) interventions, other than VBI, can be employed to further improve adherence to the ABCDE approach. Possible interventions include: alternative learning strategies, self-efficacy training, video review sessions, use of pocket cards, decision support tools, and/or augmented reality devices [6]. By conducting more research on the ABCDE approach, this algorithm may become evidence-based instead of consensus-based.

## Conclusions

This study evaluated the adherence of neonatal healthcare professionals to the ABCDE approach during simulated NALS scenarios in relation to the method of instruction. Overall adherence was rather low: participants adhered to less than a third of the ABCDE algorithm. Adherence was significantly better –both overall and regarding the individual domains of the algorithm– when the ABCDE approach was taught with video-based instruction (VBI) instead of a conventional lecture (CL). Ward clinicians demonstrated better adherence than nurses, irrespective of the instruction method. This study emphasizes the need for continuing education of the ABCDE approach and shows that VBI may be used to improve the adherence to the approach.

## Supplementary Information

**Additional file 1.** Text document, Participant characteristics

**Additional file 2.** Text document, Assessment tool and scoring instructions

## Data Availability

The datasets used and analyzed during the current study are available from the corresponding author on reasonable request.

## Abbreviations

ABCDE: Airway, Breathing, Circulation, Disability, Exposure
ANOVA: Analysis of variance
APLS: Advanced Pediatric Life Support
CL: Conventional lecture
ICC: Intraclass correlation coefficient
IQR: Interquartile range
NALS: Neonatal Advanced Life Support
NICU: Neonatal intensive care unit
NP: Nurse practitioner
PA: Physician assistant
SBT: Simulation-based training
SD: Standard deviation
VBI: Video-based instruction
