# Supplementary material for: Adherence to the ABCDE approach in relation to the method of instruction: a randomized controlled simulation study
Source: BMC Emerg Med. 2021 Oct 15;21:121. doi: 10.1186/s12873-021-00509-0 (PMC8517297; doi:10.1186/s12873-021-00509-0)
Supplement: Supplementary file 1 — Additional file 1. Text document, Participant characteristics [file 12873_2021_509_MOESM1_ESM.pdf]

Additional file 1 Participant characteristics

**ID**					
**Date**					
**Scenario (clinical problem)**					
**Scenario number**	1	2	3	4	5
**Function**	Nurse	Resident		Fellow/neonatologist	NP/PA
**Age**					
**Sex**	Female			Male	
**Previous participation in NALS training**	1x	2x		3x	≥ 4x
**Previous participation in relevant courses**	NLS/ NALS	APLS		EPLS	Other……
**Experienced real-life neonatal resuscitations**	0	1-2	3-5	6-9	≥ 10
**Working experience in pediatrics**	1-2 years	3-5 years		6-9 years	≥ 10 years
**Working experience at neonatal IC or HC**	≤ 1 month	2 – 4 months		5 months – 2 years	> 2 years
**Working experience at pediatric IC or HC**	≤ 1 month	2 – 4 months		5 months – 2 years	> 2 years
**Additional relevant working experience or training**					
**Study group**	Lecture		Video-based instruction		

Legend

NP, nurse practitioner; PA, physician assistant; NALS, neonatal advanced life support; NLS, neonatal life support; APLS, advanced paediatric life support; EPLS, European paediatric life support; IC, intensive care; HC, high care.
