# Supplementary material for: Adherence to the ABCDE approach in relation to the method of instruction: a randomized controlled simulation study
Source: BMC Emerg Med. 2021 Oct 15;21:121. doi: 10.1186/s12873-021-00509-0 (PMC8517297; doi:10.1186/s12873-021-00509-0)
Supplement: Supplementary file 2 — Additional file 2. Text document, Assessment tool and scoring instructions [file 12873_2021_509_MOESM2_ESM.pdf]

Additional file 2 Assessment tool and scoring instructions

**Domain**<br>Subdomain	**Item**	**Assessable**	**Score**		
**AIRWAY (A)**					
Airway patency	'Look-listen-feel'<br>or other adequate evaluation	Yes / No	0	1	2
**BREATHING (B)**					
Effort of breathing	Groaning	Yes / No	0	1	2
	Nasal flaring	Yes / No	0	1	2
	Retractions	Yes / No	0	1	2
	Abnormal breath sounds	Yes / No	0	1	2
	Accessory muscle use	Yes / No	0	1	2
	Respiratory rate	Yes / No	0	1	2
Efficiency of breathing	Chest auscultation	Yes / No	0	1	2
	Oxygen saturation	Yes / No	0	1	2
	Symmetric chest rise	Yes / No	0	1	2
Effect on other organs	Color	Yes / No	0	1	2
**CIRCULATION (C)**					
Circulation assessment	Heart rate	Yes / No	0	1	2
	Blood pressure	Yes / No	0	1	2
	Capillary refill time	Yes / No	0	1	2
	Pulsations	Yes / No	0	1	2
Effect on other organs	Peripheral temperature	Yes / No	0	1	2
	Urinary output	Yes / No	0	1	2
**DISABILITY (D)**					
Neurological evaluation	AVPU-score	Yes / No	0	1	2
	Pupils	Yes / No	0	1	2
	Tone	Yes / No	0	1	2
	Seizures	Yes / No	0	1	2
	Glucose	Yes / No	0	1	2
**EXPOSURE (E)**					
Temperature and skin	Body temperature	Yes / No	0	1	2
	Skin abnormalities	Yes / No	0	1	2

**RESULT**	
Score	Points
Maximum achievable score [1]	Points
Percentage score [2]	%

AVPU, alert, verbal, pain, unresponsive.

[1] Maximum achievable score is the sum of all assessable items.

[2] Percentage score is the score, divided by the maximum achievable score, multiplied by 100%.

Scoring instructions

General statements

1. This tool is based on the Dutch Advanced Paediatric Life Support manual, and was designed to assess the adherence of individual providers to the ABCDE algorithm during (simulated) newborn (and pediatric) advanced life support scenarios.
2. Score 0 = **neither** fully correct **nor** assessed at the right moment.

   Score 1 = partly correct **or** correct, but not assessed at the right moment.

   Score 2 = fully correct **and** assessed at the right moment.

   With regard to the right moment: as long as the provider maintains the sequence of the ABCDE approach, 2 points are awarded for correctly performed items. **Within one domain**, the sequence of the items does not matter. If a **domain** is skipped, a maximum of 1 point can be scored for all remaining items. When a provider initially skips an **item,** moves on to the next domain, but assesses that item correctly at a later moment, 1 point is allocated for that item. After such a short 'redemptive flashback', 2 points can be scored for remaining items.
3. Although this tool can be used to score the initial assessment and re-assessments following interventions, only the initial assessment was scored in our study. When the initial assessment is interrupted for an urgent intervention (e.g. airway opening manoeuvre, starting oxygen, fluid or glucose bolus), but adequately continued afterwards, the provider can obtain the maximum score (2 points) for the remaining items.
4. In the simulation setting, instructors should not change vital parameters when the provider performs the ABCDE approach for practical reasons. If vitals do change, the provider has to re-assess from the A. In that case, scores for already assessed items are not changed, and items that were initially not assessed, but evaluated during the re-assessment are awarded with a maximum of 1 point. From the point where the provider interrupted the initial assessment, a maximum of 2 points can be scored again. Penalty points should be considered if a provider fails to re-assess, but this situation did not occur in our study, since we kept vitals stable during the initial assessment.
5. Items are labelled as 'not assessable' when providers are not able to assess items, because they consider the situation too urgent to continue the ABCDE approach. For example, if the nurse thinks

that immediate back-up assistance of a resident is necessary in case of an obstructed airway or absence of spontaneous breathing, he/she may summon the resident first instead of continuing with the C, D, and E. The provider must clearly state that priority is given to extra help when he/she decides not to finish the ABCDE approach first. In the interval between the call and arrival of the resident, the nurse should continue the ABCDE assessment. If this time interval is not used for the ABCDE assessment, the nurse scores 0 points for **all** remaining items. If the nurse continues the ABCDE assessment, but he/she can only evaluate a few items before the resident arrives, those items are ‘assessable’, and the remaining items are ‘not assessable’, unless the resident allows the nurse to finish his/her ABCDE assessment. If two nurses start the scenario, one of them should continue the ABCDE assessment, while the other calls for help. These principles also apply to the call for back-up assistance of the fellow or neonatologist made by the resident.

6. Scoring of the provider(s) starting the scenario begins once the condition of the patient changes. So, when the nurse routinely inquires about certain parameters, while the patient is still stable, this is not scored yet. Also, remarks concerning the ‘presenting symptom(s)’ (e.g. ‘I notice that our patient becomes pale and starts to groan’) are not part of the formal ABCDE assessment, which usually starts thereafter. These remarks should not be scored (as out of sequence).
7. **All** items have to be assessed **aloud** by the providers, otherwise 0 points are allocated.
8. During the ABCDE assessment, the principle of a ‘silent cockpit’ should be applied.
9. Participants are not allowed to assume findings (e.g. “I think the color is normal”). This should be asked explicitly. Otherwise, 0 points will be awarded.
10. Providers who are called for back-up assistance (i.e. residents, fellows/neonatologists) should perform a complete ABCDE assessment upon arrival, even if they were just acquainted with the patient’s status by their colleague.
11. The provider who is being assessed is responsible for a complete and systematic ABCDE approach. When team members assess items, the provider is not awarded with points, unless he/she (a) explicitly asked those team members to do so, (b) repeats the items aloud, or (c) takes account of these items in another obvious way.

12. For items with a numerical value (i.e. respiratory rate, oxygen saturation, heart rate, blood pressure, capillary refill time, urinary output, glucose, and body temperature), an ‘interpretation’ of the value is considered correct (e.g. the infant is tachypnoeic, hypotensive, or hypoglycemic).
13. In line with daily practice and in consideration of the user-friendliness of our assessment tool, we decided not to score certain *effects on other organs* under the corresponding subdomains, because these effects are already represented as items elsewhere in the algorithm. That is, heart rate and level of consciousness should also be assessed as effects on other organs in domain B. However, these items are already part of domain C and D, respectively. In addition, respiratory rate and level of consciousness should be evaluated as effects on other organs in domain C, but these items are also assessed in domain B and D, respectively. If a provider nonetheless chooses to mention these items as effects on other organs, this is, of course, not considered to be out of sequence.

Instructions for specific items

1. *Airway patency*: the provider has to describe the airway (i.e. open, partially obstructed, obstructed, secured), including the findings on which his/her conclusion is based (e.g. the infant cries, is intubated, or has a stridor). Without the latter corroboration, a maximum of 1 point is awarded.
2. *Abnormal breath sounds:* sounds perceivable without the use of a stethoscope, such as a stridor or wheezing. Note: if ‘stridor’ is already mentioned in describing the airway, then points may automatically be awarded for this item as well.
3. *Retractions:* subcostal, intercostal, **and/or** supraclavicular/suprasternal.
4. *Use of accessory muscles of respiration:* use of the sternocleidomastoid muscle or head bobbing.
5. *Chest auscultation:* assessment of air entry on **both sides** of the thorax.
6. *Chest rise:* providers should ask/look for **symmetrical** chest excursions. If not, a maximum of 1 point is allocated.
7. *Capillary refill time:* fully correct if performed on sternum, partly correct if performed elsewhere.
8. *Pulsations:* fully correct when the central (**and** peripheral) pulsations are assessed, partly correct if only the peripheral pulsations are assessed. Central pulsations in a newborn can be felt at the bracchial artery **and/or** the femoral artery. It is allowed to assess the pulsations unilaterally.

9. *Peripheral temperature:* officially the difference between central and peripheral temperature. However, assessment of the temperature of both hands **and** feet (e.g. warm, tepid, or cold) is also fully correct. A maximum of 1 point is awarded, if the provider only assesses the temperature of the hands **or** feet, or asks for the 'temperature of the extremities' without specification.
10. *Urinary output:* produced in the last hours/shift/day. A question like 'When was the last time that this infant urinated?' is fully correct as well.
11. *AVPU-score:* if the patient does not respond when spoken to or touched, a pain stimulus must be administered to determine the AVPU-score. If not performed, a maximum of 1 point is awarded.
12. *Pupils:* size **and** light reactivity must be assessed for **both** eyes to obtain 2 points.
13. *Tone:* e.g. decerebration, decortication, opisthotonus, abnormal posture, hyper- or hypotonia.
14. *Skin abnormalities:* e.g. petechiae, hematoma, wounds, edema, or abnormal abdominal appearance.
